# Redesigning a Fidelity Measurement Tool for Early Psychosis Services: Responding to Evolving Service Landscapes in Australia

**DOI:** 10.1111/eip.70263

**Published:** 2026-09-24

**Authors:** Carly S. Talarico, Shona Francey, Craig Hamilton, Kirsten Cleland, Vidhi Oberoi, Vanessa Skellern, Yong Ly, Alyssa Milton, Ellie Brown

**Affiliations:** ^1^ Centre for Youth Mental Health The University of Melbourne Parkville Victoria Australia; ^2^ Orygen The National Centre of Excellence in Youth Mental Health Melbourne Victoria Australia; ^3^ Headspace National Youth Mental Health Foundation Melbourne Victoria Australia; ^4^ Faculty of Medicine and Health University of Sydney Sydney New South Wales Australia; ^5^ ARC Centre of Excellence for Children and Families Over the Life Course Australia

**Keywords:** early intervention in psychosis, fidelity, first episode psychosis, human centred design, learning health system, service improvement

## Abstract

**Introduction:**

In Australia, early implementation of headspace Early Psychosis (hEP) services was supported by fidelity assessments using the Early Psychosis Prevention and Intervention Centre (EPPIC) Model Integrity Tool (EMIT). Its suitability became limited by ceiling effects and limited sensitivity to evolving service contexts. This paper describes the redevelopment of EMIT into the Revised‐EMIT (REMIT) and testing implementation of the tool.

**Methods:**

A multi‐phase mixed‐methods, human‐centred design approach was used. Phases included reviewing international fidelity frameworks, stakeholder co‐design and iterative redevelopment of fidelity items, scoring and data sources. Testing of the REMIT occurred over 2 years via face‐to‐face visits to hEP clusters, using semi‐structured interviews, review of organisational documents and clinical file audits. Routine data reporting via Tableau embedded Learning Health System principles within the process. National fidelity trends across fidelity item outcomes for all six hEP service clusters were descriptively analysed.

**Results:**

The REMIT was found to reduce ceiling effects and improve sensitivity. National mean REMIT score increased from 3.99 (2023) to 4.19 (2024), with notable gains in caseload management, physical health monitoring and measuring key outcomes. Relational domains (such as continuous engagement and family involvement) remained persistently low across services and years.

**Conclusion:**

The REMIT offers a pragmatic, sensitive fidelity measure for contemporary hEP services. Findings support the use of REMIT as an active learning system rather than a static compliance mechanism. Psychometric evaluation of the tool is now required, and ongoing iterative refinement will be essential to maintain relevance within Australia's evolving youth mental health system.

## 1 Introduction

Epidemiological data consistently indicate that 75% of adults with psychiatric disorders first experience symptoms in their early teenage years and peak by the age of 24 years (Hughes et al. 2014; McGorry et al. 2007). This is compounded by a marked reluctance in the 15–25 age range to seek help, especially professional help (Rickwood et al. 2019). Targeting this early stage of disorder led to the introduction of early intervention services for psychosis globally, which has grown to become one of the most significant developments in mental health care, with a strong meta‐analytic evidence base (Addington et al. 2018; Malla and Kinkaid 2019). Early psychosis interventions aim to improve the health outcomes and functional recovery of individuals experiencing a first episode of psychosis (FEP), or those at risk of psychosis, known in Australia as being at ultra‐high risk (UHR) status (Addington et al. 2018). They ideally involve a multidisciplinary team providing multimodal treatments, including psychosocial and pharmacological interventions (Correll et al. 2018; Williams et al. 2026).

In Australia, early intervention for psychosis (EIP) services are delivered in both state‐based services (typically by hospital networks) and Commonwealth government‐funded services. The latter funding supports the delivery of EIP services via Australia's National Youth Mental Health Foundation, known as headspace, which are co‐located with primary care headspace. In 2014, the ‘headspace Early Psychosis’ (hEP) program was implemented as a cluster model (Williams et al. 2021) and designed in accordance with the Early Psychosis Prevention and Intervention Centre (EPPIC) model (Addington et al. 2018; Hughes et al. 2014). If young people are assessed as eligible for the program, they are allocated to either the FEP stream or UHR stream of care. In line with international practices within EIP services (Addington 2021) ensuring adherence to the core elements of an evidence‐based model of care (the EPPIC model) was critical to the potential success of these new services, and hEP service clusters were mandated to undergo an annual fidelity assessment process (Williams et al. 2021).

Fidelity assesses the extent to which a service delivers care as intended and evaluates the overall quality and consistency of care delivery, a process widely applied across settings that implement structured service models (Addington 2021). Ongoing fidelity monitoring is essential to sustain effectiveness in the real‐world setting (Malla and Kinkaid 2019) with retrospective (and ideally ongoing) assessments undertaken (Durbin et al. 2019; Melau et al. 2019). The original fidelity tool used for this process was known as the EPPIC Model Integrity Tool (EMIT) (Williams et al. 2021). The EMIT included 16 components comprised of 80 items which were based on the core components of the EPPIC model and scored using a combination of binary yes/no and a five‐point Likert‐scale rating system, with each item receiving a score between one (indicating no implementation) to five (indicating complete implementation/adherence to the model). Feedback on adherence to fidelity was provided by Orygen to hEP service clusters following the assessment via a comprehensive report that was also provided to commissioning agencies and the Australian Government department funding the services.

In the early stages of implementing and establishing the hEP program nationally, the EMIT was instrumental in ensuring standardised service delivery and providing consistent benchmarks across sites (Williams et al. 2021). Many of its components focused on early implementation and infrastructure requirements. However, as services matured, limitations of the tool became increasingly apparent. Whilst fidelity monitoring is essential for maintaining model integrity in early psychosis services, tools can be static and focused on structural and organisational aspects of service delivery (e.g., staffing, processes and service components), limiting their capacity to reflect evolving models of youth‐centred care. In particular, the EMIT lacked flexibility to capture emerging complexities in service delivery and contemporary components such as youth co‐design, expanded peer work and functional recovery approaches. This made it prone to ceiling effects, whereby services quickly achieved near‐maximum scores, limiting sensitivity to further improvement (Williams et al. 2021). As services mature, fidelity measurement must move beyond implementation assessment towards assessing more nuanced aims such as service quality, adaptability and sustained delivery in complex real‐world settings. Innovation in data collection within healthcare services also occurred during this timeframe, with the evolution of the concept of a Learning Health System (LHS) (Heinssen and Azrin 2022). A LHS functions cyclically, where data are collected (typically through routine clinical care) and presented back to clinicians and service leads, who then use it for evaluation and improving practice (Kilbourne et al. 2025).

In response to changes in the service landscape, a multi‐phase, collaborative revision process occurred to develop a tool that moved fidelity assessment from static measures of implementation outcomes towards a more dynamic evaluation of implementation quality in real‐world service delivery. This intended to ensure that model fidelity aligned more closely with current service delivery whilst emphasising quality assurance, adherence to clinical standards and continuous improvements in service provision.

The first aim of this paper was to outline the multi‐phase redevelopment of the EMIT into the REMIT—‘Revised EPPIC Model Integrity Tool’—including describing the structural changes in data collection and scoring design. The second aim was to test the implementation of the tool by presenting preliminary outcomes of utilising the revised tool (REMIT) as a measure of fidelity and service improvement across Australian hEP service clusters over 3 years.

## 2 Methods

### 2.1 Participants and Setting

Participants included stakeholders from the six hEP service clusters across Australia located in Adelaide, Darwin, North Perth (three sites—Joondalup, Osborne Park and Midland), Southeast Queensland (two sites—Meadowbrook and Southport), Western Sydney (three sites—Parramatta, Penrith and Mount Druitt) and Southeast Melbourne (four sites—Bentleigh, Narre Warren, Frankston and Dandenong). Specifically, this included hEP clinical leaders, operations managers, data managers, functional recovery workers, peer support workers and young people receiving care from the service along with their family members and carers.

### 2.2 Study Design

The conceptual framework that guided this project drew on human‐centred design (HCD) principles. HCD is a structured theoretical framework underpinned by iterative co‐design, stakeholder engagement and user‐centred problem solving (Coetzer‐Liversage and Stein 2025; Göttgens and Oertelt‐Prigione 2021; Zhai et al. 2025). It complements traditional implementation science principles underpinning fidelity assessment that focus on utilising mixed‐methods (i.e., qualitative and quantitative methodologies) to bridge the ‘knowledge to practice’ gap. Utilising HCD principles thus supported the redesign process to reflect real‐world service workflows and stakeholder experiences. Methodologies utilised included reviewing existing fidelity frameworks and iterative stakeholder co‐design consultation sessions. The redesign process from EMIT to REMIT took place over 12 months, between December 2021 and January 2023, with all consultations carried out online due to the COVID‐19 pandemic. REMIT outcome data were collected between April 2025 and June 2025.

### 2.3 Multi‐Phase Development of the REMIT

#### 2.3.1 Phase 1—Review of Existing EMIT and International Fidelity Frameworks

A review of EMIT's core components via past reports was conducted to identify recurring limitations, ceiling effects and areas lacking sensitivity to change. Relevant frameworks, key reports and general resources were reviewed, with a complete outline listed in Table 1 (Addington 2021; Chandra et al. 2018; Commonwealth of Australia 2010; Ernst and Young 2020; Orygen, n.d.; Stavely et al. 2013). Academic literature, including a systematic review of essential components of FEP services were also revised (Addington et al. 2013; Milton et al. 2022). The review phase focused on identifying limitations of the existing EMIT and other tools and frameworks used internationally, such as usability issues and contextual factors that limited tool effectiveness. These insights formed the foundation for the second phase of the redevelopment process.

**TABLE 1:** International clinical practice guidelines, standards and models reviewed.

Name of resource	Publication year	Country of relevance
The National Standards for Mental Health Services	2010	Australia
EPPIC model and service implementation guide	2013	Australia
National Clinical Audit of Psychosis (NCAP) resources	2017–ongoing	United Kingdom
Orygen key principles underpinning youth mental health models	2018	Australia
Standards for early intervention in psychosis services – 1st edition	2018	United Kingdom
Evaluation of the early psychosis youth services programme – final report	2020	Australia
First Episode Psychosis Services Fidelity Scale (FEPS‐FS) and Manual	2021	Canada

#### 2.3.2 Phase 2—Stakeholder Co‐Design

A series of five semi‐structured consultation sessions were held with 18 key stakeholders from the six hEP service clusters. Each site's operations manager, clinical lead and data manager were present to ensure representation of both clinical and operational perspectives with two youth peer workers consulted separately. Service‐users (young people) and their family members were not consulted at this stage. Sessions were led by Orygen fidelity researchers and focused on reviewing the relevance, feasibility and clarity of the proposed changes to fidelity items and data collection procedures that came from Phase 1. Decision‐making occurred via consensus, and when required, further input was gathered from experts in specific content areas (such as cognition and peer work), drawing on both clinical expertise and existing literature (Thomas et al. 2023; Zbukvic et al. 2025). This phase mirrors work carried out by Hyam et al. (2026) whereby academics, clinicians, and individuals with lived experience informed the planning and development of a fidelity tool.

#### 2.3.3 Phase 3—Redevelopment of the Fidelity Tool

##### 2.3.3.1 Phase 3a. Revising Fidelity Core Components and Items

This phase involved refining the tool's structure and content using the findings from Phase 1 and 2. Drawing on feedback from stakeholders, REMIT components and items were amended to ensure they remained: (a) relevant to early psychosis care, (b) supported by evidence, and (c) achievable across evolving hEP services and governance arrangements.

##### 2.3.3.2 Phase 3b. REMIT Data Collection Procedures

Fidelity data infrastructure was also improved. Since the EMIT was originally designed, a routinely collected hEP Minimum Data Set (hEP MDS) comprising clinical, demographic, service and outcome data had been introduced across services. Evolution of the capabilities of this data system (known as the headspace Application Platform Interface, ‘hAPI’) allowed for more comprehensive data extraction from hEP service clusters that directly mapped onto REMIT core components. Data captured within hAPI were reported through Tableau, a visual analytics platform that provided standardised fidelity reports and performance metrics across hEP service clusters. These updates enabled the retrieval of real‐time data within defined reporting periods that aligned to the fidelity assessment period, ensuring more accurate fidelity scoring and demonstrating the strength of integrating a LHS with fidelity assessments (Addington et al. 2024).

##### 2.3.3.3 Phase 3c. REMIT Scoring Framework

A review of international fidelity measures was undertaken (Addington 2021; Lloyd‐Evans et al. 2016) to understand best practice in fidelity scale scoring. Unlike weighted scoring approaches that aggregate fidelity into broad categories (Bond and Drake 2020), REMIT adopted a simple unweighted scoring system to support transparency and ease of interpretation (Addington 2021; Hyam et al. 2026) and improve sensitivity to incremental changes in fidelity over time (Bond and Drake 2020). As such, all binary yes/no scoring was replaced with five‐point Likert scales (used only for some EMIT items) for all REMIT fidelity items (Bond and Drake 2020).

Item scores were used to generate a total combined REMIT fidelity score (mean and standard deviation [SD]), ranging from 1.00 to 5.00. Scores of 1.00–3.00 indicated poor adherence/need for improvement, 4.00 indicated good adherence and 5.00 indicated excellent adherence to the EPPIC model.

### 2.4 REMIT Process Testing

The revised tool was launched in 2023, with fidelity assessments conducted annually at each of the six hEP service clusters across Australia since its implementation. Face‐to‐face visits to sites occurred over 2 days with interviews and data collection conducted by two Orygen researchers, who completed analysis and reporting using a consensus‐checking approach.

It was anticipated that the first REMIT fidelity assessment would identify a greater number of areas for improvement than previous assessments conducted with the EMIT, reflecting revisions to the scoring, items and components to reduce ceiling effects. This anticipated variation in fidelity scores was intended to provide more actionable information to inform quality improvement activities and ongoing training initiatives—which in turn would lead to improved fidelity scores over time, thus acting as a LHS continuous feedback loop that improves future healthcare delivery. The complete REMIT developed and used in this project can be found in Data S1.

#### 2.4.1 Analysis

To test the implementation of the tool, longitudinal trends in national fidelity scores for the six hEP service clusters were examined by Orygen fidelity researchers. Data are presented for the last two completed REMIT assessment cycles which occurred in 2023 and 2024 calendar years. Analyses were limited to descriptive statistics, including means, SDs and year‐to‐year change scores (calculated as the 2024 mean minus the 2023 mean), where positive values indicated an increase in fidelity scores, negative values indicated a decrease and 0.00 indicated no change.

The utility of the MDS information captured via the hAPI/Tableau platform was explored through establishing a baseline of item scores from the year before Tableau reports were made available (2022), followed by the two consecutive calendar years (2023–2024) where results for each fidelity item measurable via hAPI were extracted and presented as percentages.

### 2.5 Ethics

Redesign was part of a service improvement project and did not require ethics approval. Recurrent REMIT visits have ethics approval via the Royal Melbourne Hospital HREC (reference number: HREC/87399/MH‐2022) along with site‐specific governance processes. Client‐facing materials such as Participant Information and Consent Forms (PICFs) and interview schedules were revised and updated in close collaboration with youth peer support workers and youth representatives from Orygen's Youth Reference Group (YRG) to ensure relevance and appropriateness of language.

## 3 Results

### 3.1 Multi‐Phase Development of the REMIT

An overview of the key modifications made during the redevelopment of EMIT to REMIT is displayed in Table 2. Whilst the overall fidelity domains remained largely consistent, several components were consolidated, refined or removed to better reflect contemporary early psychosis service delivery. Examples of fidelity items which changed from EMIT to REMIT can be found in Table S1, along with the complete EMIT and REMIT tools discussed throughout. The number of components was reduced from 16 to 11, and the number of items comprising those components was also reduced from 80 to 32 items to further streamline the fidelity process. Three EMIT components (access to inpatient care, subacute beds and interagency partnerships) were removed as co‐design consultations found these elements to have limited utility in assessing fidelity within a primary care‐based early psychosis model.

**TABLE 2:** Comparison of EMIT components to REMIT components.

	EMIT	REMIT
Components	Community education and awareness Early detection and care of UHR young people Easy access to services Home‐based assessment and care Access to streamed youth‐friendly inpatient care Access to youth‐friendly sub‐acute beds Case management Medical interventions Psychological interventions Functional recovery programme Group programmes Family programmes and family peer support Youth participation and peer support Mobile outreach Partnerships Workforce development	Community education and awareness Early detection and care of UHR young people Home‐based assessment and care 3a. Easy access to services 4 Case management 5 Medical treatments 6 Psychological interventions 7 Functional recovery programme 7a. Group programmes 8 Family programmes 9 Youth Participation 10 Peer support (and family peer support) 10 Mobile outreach 11 Workforce development
Items	80 items	32 items
Scoring Framework	Binary yes/no Five‐point Likert scale (1–5)	Five‐point Likert scale (1–5)
Data Sources	Semi‐structured interviews with hEP staff Reviewing operational policies and guidelines Reviewing routine service activity and clinical data collected and managed by headspace National (hN)—referred to as ‘hAPI data’	Semi‐structured interviews with young people, families, carers, and hEP staff Reviewing operational policies and guidelines Reviewing hAPI data File audits of 10 randomly selected young people (7 FEP, 3 UHR)

In addition, the REMIT expanded data collection to include semi‐structured interviews with young people and family members/carers of those who had used the service, as well as file audits of 10 randomly selected young people via electronic medical records (EMRs) (Ritter et al. 2001). This inclusion was driven by consistent feedback from hEP service clusters recognising the need for broader perspectives in service evaluation outside of senior staff members and service users, alongside increasing international consensus of the involvement of lived experience voices at all levels of youth mental health service delivery (Killackey 2023).

The REMIT scoring framework was applied across all fidelity items, generating overall fidelity scores on a five‐point scale. This change enabled services to demonstrate implementation quality, offering clearer feedback on performance gradients and distinguishing more clearly between partial and full adherence to the EPPIC model (Addington 2021; Bond and Drake 2020; Hyam et al. 2026).

### 3.2 REMIT Process Testing

To test the utility of the re‐designed tool as a measure of model fidelity and service improvement support, data were collected across 2 years of service delivery (2023 and 2024). Gradual improvements in fidelity scores were observed across multiple domains during the evaluation period (Tables 3 and 4), suggesting the revised scoring framework was sensitive to changes in service delivery and implementation progress.

**TABLE 3:** Key fidelity items assessed using hAPI data^a^ displayed via the Tableau platform (national averages extracted for 2022–2024 calendar years).

REMIT item	2022	2023	2024
1: DUP measured for all suspected FEP	69.1% (*n* = 311)	71.3% (*n* = 354)	84.1% (*n* = 397)
3: Varied referral sources	Top 5: 1. Inpatient (27.5%, *n* = 239) 2. Community mental health service (18.9%, *n* = 164) 3. Primary health care (11.9%, *n* = 103) 4. Family/friend (9.6%, *n* = 83) 5. headspace (9.2%, *n* = 80)	Top 5: 1. Inpatient (27.8%, *n* = 276) 2. Community mental health service (19.5%, *n* = 194) 3. headspace (10.9%, *n* = 108) 4. Primary health care (8.6%, *n* = 85) 5. Self‐referred (8.4%, *n* = 83)	Top 5: 1. Inpatient (27.8%, *n* = 262) 2. Community mental health service (19.1%, *n* = 180) 3. headspace (16.2%, *n* = 153) 4. Family/friend (7.9%, *n* = 75) 5. Self‐referred (6.4%, *n* = 60)
4: Initial assessments conducted face‐to‐face (FEP within 3 days, UHR within 5 days)	UHR 42.2% (*n* = 177) FEP 38.9% (*n* = 175)	UHR 46.0% (*n* = 234) FEP 42.4% (*n* = 205)	UHR 53.6% (*n* = 253) FEP 36.4% (*n* = 172)
11b: Service drop‐outs	7.7% (*n* = 61)	9.6% (*n* = 78)	9.2% (*n* = 67)
12a: YP assessed/reviewed every 90 days	26.7% (*n* = 1259)	35.0% (*n* = 1501)	45.8% (*n* = 1908)
12b: Tenure of care (FEP at least 2 years of care, UHR at least 6 months)	UHR 81.3% (*n* = 434) FEP 31.8% (*n* = 172)	UHR 69.6% (*n* = 321) FEP 34.6% (*n* = 202)	UHR 69.4% (*n* = 284) FEP 33.2% (*n* = 147)
12c: YP seen face‐to‐face at least monthly	37.8% (*n* = 855)	43.3% (*n* = 942)	49.1% (*n* = 1020)
13a: YP reviewed by a doctor within 72 h for FEP or 2 weeks for UHR	UHR 49.2% (*n* = 206) FEP 48.0% (*n* = 216)	UHR 51.5% (*n* = 262) FEP 49.8% (*n* = 241)	UHR 55.7% (*n* = 263) FEP 58.5% (*n* = 276)
13b: YP reviewed by a psychiatrist within 14 days	45.6% (*n* = 396)	49.3% (*n* = 490)	52.0% (*n* = 491)
14: FEP received antipsychotic medication by first 90 day review	72.4% (*n* = 249)	74.4% (*n* = 323)	81.9% (*n* = 303)
17: Physical health screening/monitoring offered/carried out every 90 days	49.8% (*n* = 925)	60.8% (*n* = 1059)	65.4% (*n* = 1064)
19: CBT provided for symptoms and comorbidities	53.2% (*n* = 572)	45.1% (*n* = 471)	58.5% (*n* = 498)
24a: Families contacted by a clinician within 48 h of entry to service and provided psychoeducation	42.7% (*n* = 371)	42.6% (*n* = 423)	51.4% (*n* = 485)
24b: Families contacted once every 90 days by any service provider	28.6% (*n* = 532)	32.5% (*n* = 566)	31.4% (*n* = 510)
24c: Families contacted by fPSWs within 7 days	42.9% (*n* = 461)	37.3% (*n* = 389)	39.0% (*n* = 332)
32a: % of YP assessed using CAARMS	UHR 78.5% (*n* = 329) FEP 87.8% (*n* = 395)	UHR 82.9% (*n* = 422) FEP 91.5% (*n* = 443)	UHR 89.8% (*n* = 424) FEP 93.0% (*n* = 439)
32b: % of YP with CAARMS completed at every 90 day review	48.8% (*n* = 340)	57.7% (*n* = 345)	65.0% (*n* = 367)

Abbreviations: CAARMS: the Comprehensive Assessment of At‐Risk Mental States; CBT: cognitive behavioural therapy; DUP: duration of untreated psychosis; FEP: first‐episode of psychosis; fPSWs: family peer support workers; UHR: ultra‐high risk of psychosis; YP: young people.

^a^ In accordance with REMIT scoring guidelines, item scores of ≥ 80% indicated full adherence, 50%–79% indicated partial adherence, and ≤ 49% indicated an item as unmet. Exceptions to this standard existed for two items: item 3 (number of referral sources) where high fidelity was defined as five or more referral sources; and item 11b (service drop‐outs) where a drop‐out rate of ≤ 10% indicated high fidelity.

**TABLE 4:** Combined mean and standard deviation of REMIT fidelity items for six hEP service clusters for 2 years.

REMIT item	2023		2024		Change^a^
	Mean	SD	Mean	SD	
1: DUP measured for all suspected FEP	4.17	1.21	4.50	0.50	+0.33
2: Case detection and promoting referrals	4.67	0.75	5.00	0.00	+0.33
3: Varied referral sources	5.00	0.00	5.00	0.00	0.00
4: Initial assessments	3.17	0.69	3.33	0.47	+0.16
5: Flexible delivery of care^b^	4.00	0.82	4.33	0.47	+0.33
6: Variety of interventions delivered	4.83	0.37	5.00	0.00	+0.17
7: Caseload per cluster meets targets	2.83	0.69	4.17	1.21	+1.34
8: Engagement with case manager	3.17	1.46	2.83	1.46	−0.34
9: ITP reviewed every 90 days	4.33	1.11	4.33	0.75	0.00
10: Incomplete recovery identified at 6mths	4.00	1.53	4.50	0.50	+0.50
11: Service drop‐outs	4.33	0.75	4.67	0.47	+0.34
12: Continuous engagement encouraged	1.00	0.00	1.17	0.37	+0.17
13: Medical review^c^	3.17	1.21	3.00	1.00	−0.17
14: Antipsychotic medication dosing prescribed within guidelines	4.83	0.37	5.00	0.00	+0.17
15: Clozapine used for medication resistance	4.33	1.49	5.00	0.00	+0.67
16: Physical health supported	5.00	0.00	4.67	0.47	−0.33
17: Physical health screening and monitoring	2.17	1.21	3.50	0.96	+1.33
18: Psychological treatment goals reviewed	4.83	0.37	5.00	0.00	+0.17
19: CBT provided	3.00	0.58	3.33	0.47	+0.33
20: Cognitive/neuropsychological assessments	4.50	1.12	4.67	0.47	+0.17
21: Functional recovery goals documented	5.00	0.00	5.00	0.00	0.00
22: Functional recovery groups run^d^	5.00	0.00	4.33	1.49	−0.67
23: System to identify intensive mobile outreach need	5.00	0.00	4.33	1.49	−0.67
24: Engagement with family/friends	1.33	0.47	1.17	0.37	−0.16
25: Family work considered	5.00	0.00	4.83	0.37	−0.17
26: Peer support workers engaged	5.00	0.00	5.00	0.00	0.00
27: Embedded youth participation	4.83	0.37	5.00	0.00	+0.17
28: fPSW/PSWs are actively engaged	4.00	1.00	5.00	0.00	+1.00
29: Staff adequately prepared/supported	5.00	0.00	4.83	0.37	−0.17
30: Staff engagement with training	2.17	0.37	3.00	0.58	+0.83
31: Multidisciplinary teams	5.00	0.00	5.00	0.00	0.00
32: UHR/FEP status determined using CAARMS	3.00	1.00	3.67	0.94	+0.67
	Overall mean = 3.99		Overall mean = 4.19		

Abbreviations: CAARMS: the Comprehensive Assessment of At‐Risk Mental States; CBT: cognitive behavioural therapy; DUP: duration of untreated psychosis; FEP: first‐episode psychosis; fPSW: family peer support worker; ITP: individual treatment plan; PSWs: peer support workers; UHR: ultra‐high risk of psychosis.

^a^ Change: 2024 mean minus 2023 mean; +: increase; −: decrease; 0.00: no change.

^b^ ‘Flexible delivery of care’ refers to the extent to which hEPs provide outreach, telehealth, after‐hours appointments and location‐flexible care according to young person needs.

^c^ ‘Medical review’ refers to review by a psychiatrist, consultant, registrar and/or general practitioner.

^d^ ‘Functional recovery groups being run’ refers to the availability and delivery of structured group interventions targeting education, employment and social functioning of young people.

Table 3 presents hAPI data from Tableau reports for key REMIT items (national averages) for the 2022–2024 calendar years. Importantly, the 2022 data displayed in Table 3 provided a baseline measure of fidelity item performance against which changes observed during the first 2 years of REMIT implementation (2023–2024) could be compared. This demonstrates the utility of enhanced hAPI data infrastructure and Tableau reporting, as it enabled extraction, visualisation and longitudinal monitoring of fidelity indicators. The availability of these objective performance data allowed service clusters to track adherence to the EPPIC model in real time and identify areas requiring targeted quality improvement efforts.

As shown in Table 3, improvements were seen in service quality metrics across the 3 years. Areas with the largest improvements included the proportion of young people in the FEP stream for whom Duration of Untreated Psychosis (DUP) was measured (item 1) increased by 15.0% (from 69.1% to 84.1%), with similar percentage increases in assessment and care planning indicators including the proportion of young people assessed or reviewed every 90 days (item 12a) increased by 19.1% (from 26.7% to 45.8%), and those seen face‐to‐face at least monthly (item 12c) increased by 11.3% (from 37.8% to 49.1%). Completing the Comprehensive Assessment of At‐Risk Mental States (CAARMS) at initial assessment (item 32a) consistently met or neared full adherence benchmarks (> 80%) across all years. There were other domains where data captured through the hAPI MDS platform highlighted challenges with model delivery and minimal improvement with the introduction of the Tableau real‐time data reports. For example, contact by family peer support workers within 7 days (item 24c) decreased from 42.9% to 39.0% across years.

Table 4 presents the mean REMIT score and SD for each item as a combined score for the six hEP service clusters in 2023 and 2024 (the first 2 years of REMIT implementation). Overall, fidelity across the six hEP service clusters was high in both years, with an increase in mean REMIT score from 3.99 in 2023 to 4.19 in 2024. Additionally, SDs were generally lower in 2024, indicating greater consistency in implementation across services.

Analysis of the specific REMIT items in Table 4 allows assessment of where ceiling and floor effects may be occurring. Several items achieved mean scores of 5.00 in both years, namely referral pathways (item 3), documentation of functional recovery goals (item 21), engagement of PSWs (item 26) and the presence of multidisciplinary teams within the hEP clusters (item 31). Other domains where mean scores were consistently high included, case detection (item 2), the delivery of varied interventions (item 6), antipsychotic medication (item 14), the review of psychological treatment goals (item 18), family work (item 25), embedding youth participation (item 27) and support for staff (item 29). Notable improvements over the years were observed in several areas. Mean scores for caseloads (item 7) increased from 2.83 to 4.17, whilst physical health screening (item 17) increased from 2.17 to 3.50. However, the standard deviations for these items were also high (SD 1.21), indicating heterogeneity in local implementation across hEP clusters. This greater variability was observed for other items relating to service delivery practices, including engagement with case managers (both years SD 1.46), medical review (2023 SD 1.21, 2024 SD 1.00), clozapine use (2023 SD 1.49) and intensive mobile outreach practices (2024 SD 1.49). In contrast, items reflecting structural components of the model—such as varied referral sources, peer workforce integration and multidisciplinary team composition—showed minimal variation across hEP service clusters and years.

Finally, some areas of the model of care scored comparatively low in both years. Items with the lowest mean scores and minimal improvement included continuous engagement (item 12: 2023 1.00; 2024 1.17) and engagement with family and friends (item 24: 2023 1.33; 2024 1.17). These scores indicate ongoing challenges in these domains of service delivery and/or measurement of fidelity to this practice.

## 4 Discussion

This study presents the findings from a program of work re‐developing and testing the implementation of a national fidelity tool used in EIP services. It presents the HCD‐informed redesign process, followed by the first 2 years of REMIT fidelity implementation outcomes across six Australian hEP service clusters.

Regarding the first aim, the HCD‐centred redesign process drew on the lived experience and expertise of key stakeholders to ensure the redevelopment reflected evolving real‐world service delivery nuances (Coetzer‐Liversage and Stein 2025). The current study found that systematically embedding stakeholder input helped foster alignment with local workflows and enhanced contextual relevance, particularly in diverse and dynamic service delivery settings with geographical and demographic differences of young people serviced between each state and territory, as well as cluster composition variations (single‐site and multi‐site clusters). This aligned with emerging evidence on balancing firm implementation adherence with flexible contextual appropriateness within fidelity tools (Albers et al. 2024; Coetzer‐Liversage and Stein 2025). There were challenges to following this process however, as co‐design demands considerable time, sustained engagement and facilitation expertise (Coetzer‐Liversage and Stein 2025). The current redesign processes mitigated these constraints by utilising five consultation sessions held at varying times, inviting stakeholders from diverse professional and lived experience backgrounds and encouraging engagement via the semi‐structured design of sessions to facilitate discussion and collaboration. The use of multiple consultation formats and stakeholder perspectives is consistent with co‐design practice, where diverse experiential and professional viewpoints strengthen relevance, acceptability and usability across complex service contexts (Milton et al. 2021; Vaughan et al. 2026).

The integration of enhanced electronic data systems (collected in hAPI and accessible via Tableau Reports) played a key role in updating the REMIT fidelity process and aligning to best practice in an era of learning health systems (Easterling et al. 2021; Heinssen and Azrin 2022; Kilbourne et al. 2025). Digital data extraction enabled improved efficiency and timeliness, and greater reporting consistency across key REMIT items. Automated extracts of hAPI data also strengthened longitudinal monitoring, providing clinical sites with clearer oversight of fidelity performance trajectories across time. Whilst evolving technology has the potential to substantially improve efficiency, it does not replace the need for human judgement and contextual interpretation (Kinkaid et al. 2025). This is particularly pertinent when interpreting results across vastly different service contexts, such as those seen between the locations of hEP service clusters. This need to balance technological efficiencies and human interpretation is reflected in several fidelity items requiring narrative explanation, situated clinical reasoning or staff reflections that cannot be captured through automated data collection alone. From reviewing existing international fidelity tool development (Lloyd‐Evans et al. 2016) and local stakeholder consultations, it was determined that quantitative indicators derived from data collected via hAPI must be interpreted in parallel with qualitative perspectives from service users (young people and families) and service providers (hEP staff) to produce nuanced implementation insights and accurate fidelity measurements (Kinkaid et al. 2025). For example, items assessing continuous engagement and outreach (item 12), and determination of UHR versus FEP status (item 32), incorporated both hAPI indicators and qualitative interview data. For these items, quantitative hAPI data were used to assess the extent of service delivery, whilst interviews provided contextual information regarding the quality and consistency of implementation. In such circumstances, fidelity ratings were determined through researcher consensus‐checking and consideration of all available evidence sources against predefined scoring criteria, enabling a more comprehensive assessment than either quantitative or qualitative data alone. We therefore argue that technology can function as a foundation for fidelity assessment in the REMIT model; however, it remains dependent on interpersonal engagement to ensure accuracy, contextual appropriateness and clinical relevance (Albers et al. 2024; Coetzer‐Liversage and Stein 2025; Kinkaid et al. 2025; Kopelovich et al. 2024).

Findings from the testing of the REMIT tool found gradual yearly improvements in fidelity performance across multiple domains in hEP service clusters nationally, in contrast to the EMIT where ceiling effects became quickly apparent (Williams et al. 2021). Certain domains (assessment and care planning and completing the CAARMS at 90‐day follow up intervals for UHR) displayed marked improvements, whilst others (initial assessments) showed more modest gains. Fidelity was consistently higher for domains that were both technically specified and structurally embedded within routine workflows (e.g., assessments, functional recovery documentation, multidisciplinary teams and peer support roles). This suggests that elements of care are well‐embedded within hEP service delivery but also may simply reflect easier objective recording processes.

In contrast, relational domains reflecting relational and engagement processes (e.g., engagement with family and friends and continuous engagement) remained comparatively lower across assessment periods. Whilst the revised fidelity tool increased visibility and assessment of family engagement activities, implementation remains dependent on factors such as workforce capacity and skills, competing service demands and family willingness to participate. The low scoring of family engagement, despite the inclusion of family interviews as a core data source in REMIT, demonstrates the value of the revised fidelity process in identifying components of care that remain challenging to implement consistently. This suggests that whilst fidelity monitoring alone may not drive practice change, it can provide important visibility of areas requiring targeted service improvement, as well as areas where performance is improving. This is consistent with qualitative findings highlighting the complexity of sustained relational continuity and collaborative involvement across service transitions (Milton et al. 2022; Powell et al. 2024). Recent focus group research with service users and families found the highest‐rated idea for improving family engagement was for service providers to act as mediators and advocates, helping service users and their families navigate difficult conversations (Muddle et al. 2024). In contrast, healthcare professionals highlighted a need for additional training in working effectively with carers and families to enhance their confidence in this area of service delivery (Muddle et al. 2024). Together, these perspectives suggest challenges in family engagement could stem from resourcing and training gaps within services, and from misunderstandings between professionals and young people and their families. It is also possible that relational care remains more difficult to standardise and operationalise within fidelity frameworks. Additionally, persistently low scores in domains such as continuous engagement and family involvement are consistent with qualitative findings highlighting the complexity of sustained relational continuity and collaborative involvement across service transitions (Milton et al. 2022; Powell et al. 2024).

Encouragingly, we observed consistently high fidelity scores for peer support and functional recovery domains which represent the more holistic domains of early psychosis care. This aligns with growing evidence that peer‐supported self‐management approaches can strengthen recovery planning, continuity of care and service engagement (Johnson et al. 2018; Milton et al. 2017, 2024, 2026; Vaughan et al. 2026). Evidence from qualitative studies suggests recovery is shaped not only by symptom change but also by social connection, identity development and meaningful participation—reinforcing the importance of functional recovery and peer support components within EIP services (Caldwell et al. 2025).

Together, the findings from the redesign and testing of a fidelity tool for services within the Australian hEP model supports the notion that fidelity assessment strengthens implementation quality and supports ongoing service improvement (Albers et al. 2024; Kopelovich et al. 2024). In better understanding the level of fidelity to a model of care, researchers are better able to discern early warning signs of implementation drift. The findings presented here demonstrate how fidelity monitoring can move beyond compliance or audit functions and become a system‐level resource. The steady increase in fidelity scores across hEP service clusters illustrates REMIT's value in fostering feedback loops, identifying areas requiring targeted intervention and informing service‐level planning (Hyam et al. 2026). As such, fidelity has become a dynamic contributor to service quality and long‐term sustainability rather than a static evaluation requirement.

### 4.1 Limitations

Several limitations should be considered when interpreting the current findings. Results are preliminary and may not reflect stable trends across hEP service clusters beyond the period reported in this study. Variability also exists in data completeness in different hEP service clusters and years and thus may not reflect on‐the‐ground clinical practice. Evolving national service delivery context means some items may require further validation or refinement to optimise clarity, usability and measurement outcomes. Additionally, this current study did not examine associations between fidelity and clinical or functional outcomes, limiting conclusions regarding the impact of improved fidelity on service effectiveness.

Further, whilst the REMIT redevelopment process incorporated extensive stakeholder consultation and co‐design principles, supporting the face validity of the revised tool, formal psychometric evaluation was beyond the scope of the current study. Thus, important measurement properties, including inter‐rater reliability, construct validity, predictive validity and sensitivity to change, were not examined. These collective limitations highlight that REMIT fidelity implementation is an ongoing and evolving process, rather than a finalised static event.

### 4.2 Future Directions

The REMIT tool is intended to be viewed as part of an evolving fidelity process in the Australian EIP context where LHS development is progressing rapidly. Future work will involve additional HCD‐informed cycles of development, including more detailed stakeholder engagement and iterative refinement of items. Opportunities exist to increase automation, for example through AI‐assisted transcription of interviews and automated file auditing. Such advancements may reduce administrative burden; however, caution should be taken to utilise such technologies safely. Expanding the REMIT beyond EIP services could also enhance its applicability to diverse youth mental health models, particularly as the youth mental healthcare ecosystem continues to evolve and expand both within Australia and internationally. Integrating fidelity data more closely with service‐level outcome performance indicators may further improve its utility as a continuous quality improvement tool. Further redevelopment cycles should continue to balance rigour and flexibility, ensuring core fidelity items remain stable whilst adapting to local service contexts (Albers et al. 2024).

## 5 Conclusion

Fidelity tools should be viewed as evolving instruments, benefiting from iterative updates rather than one‐off designs, to remain responsive to changing workflows, technology and service demands. The redevelopment of the EMIT to the REMIT demonstrates how fidelity measurement can be strengthened through HCD, enhanced data sources and scoring, and embedding within a LHS. This has resulted in more nuanced and consistent assessments of EPPIC model delivery across hEP service clusters. Importantly, the REMIT demonstrates how fidelity monitoring can function as an active learning system rather than a static compliance mechanism, enabling services to identify strengths, gaps and opportunities for ongoing improvement. Whilst improvements in national fidelity performance suggest the REMIT is supporting greater consistency and quality assurance, persistently low scores in some domains highlight where further practice change is required. Ongoing redevelopment of the REMIT should therefore focus on maintaining rigour whilst increasing flexibility, supporting continuous quality improvement, and ensuring fidelity data meaningfully informs service development in a continually evolving youth mental health landscape.

## Funding

The authors have nothing to report.

## Conflicts of Interest

The authors declare no conflicts of interest.

## Supporting information


**Data S1:** Measuring fidelity using the REMIT.

**Table S1:** Examples of fidelity items changed from EMIT to REMIT.

## Data Availability

The data that support the findings of this study are available on request from the corresponding author. The data are not publicly available due to privacy or ethical restrictions.
