# Supplementary material for: Redesigning a Fidelity Measurement Tool for Early Psychosis Services: Responding to Evolving Service Landscapes in Australia
Source: Early Interv Psychiatry. 2026 Sep 24;20(9):e70263. doi: 10.1111/eip.70263 (PMC13612145; doi:10.1111/eip.70263)
Supplement: Supplementary file 1 [file EIP-20-0-s002.docx]

**Supplementary Material**

**Table S1: Examples* of Fidelity Items which changed from EMIT to REMIT**

| **EMIT** | **REMIT** | **Reason for change** |
| --- | --- | --- |
| Item 3: Frequency of community education and awareness activities focusing on case detection and promoting referrals to the hEP service | Item 2: The hEP service carries out a comprehensive range of activities with a focus on case detection and promoting referrals | EMIT assessed the frequency of community education and awareness activities using predefined intervals (e.g., monthly, quarterly). REMIT shifted the focus to the breadth and annual volume of referral-promotion and case-detection activities, providing a more comprehensive assessment of community engagement efforts. |
| Item 11: Time to initial assessment from referral to hEP program for suspected FEP (incl. triage processes);  Item 12: Time to initial assessment from referral to hEP program for suspected UHR (incl. triage processes);  Item 16: Feedback of the assessment is provided to families unless contraindicated | Item 4: Initial assessments are conducted ASAP from date of referral to the hEP services with feedback provided to families (unless contraindicated) | EMIT assessed assessment timeliness for FEP and UHR cohorts, and family feedback, as three separate items. REMIT consolidated these indicators into a single item with two structured sub-components: (a) timely assessment for FEP and UHR referrals and (b) provision of assessment feedback to families. This reduced duplication and strengthened the assessment of early engagement processes. |
| Item 23: Time to allocation to case manager from acceptance into the FEP stream;  Item 24: Time to allocation to case manager from acceptance into the UHR stream;  Item 25: Time from allocation to first case manager contact via any means including SMS, phone, etc. (FEP and UHR) | Item 8: Engagement with case manager occurs within a timely manner from their acceptance into hEP | EMIT assessed allocation and engagement processes across three separate items. REMIT combined these indicators into a single item with two sub-components assessing (a) timely allocation to a case manager and (b) timely contact with the allocated case manager. This simplified scoring and reduced overlap between closely related processes. |
| Item 30: Incomplete recovery identified at 3 months | Item 10: Incomplete recovery identified at 6 months and triggers a specific response aimed at facilitating recovery | REMIT expanded the focus from identifying incomplete recovery to assessing whether structured clinical responses and recovery-enhancing strategies were implemented following identification of persistent symptoms or treatment resistance. |
| Item 31: Transition from service plan | Item 11: The hEP service promotes proactive engagement throughout tenure of care | EMIT focused primarily on discharge planning. REMIT broadened the construct to include proactive engagement, dropout prevention, community outreach, and preparation for discharge, reflecting a more comprehensive assessment of engagement across the episode of care. |
| Items 38-40: Access to medical review and consultant psychiatrist review | Item 13: Young person accepted onto the hEP program received medical review within an appropriate timeframe | EMIT assessed access to medical care across multiple items. REMIT consolidated these measures into a single item assessing timely access to both medical and psychiatric review, reducing redundancy and improving interpretability. |
| No equivalent EMIT items | Item 17 (physical health); Item 18-20 (psychological interventions); items 21-22 (vocational/functional recovery) | REMIT introduced several new fidelity domains to improve coverage of evidence-based interventions and recovery-oriented practice that were not explicitly assessed in EMIT. |

**Note: The examples presented are illustrative of key changes made during redevelopment of the EMIT into the REMIT and do not capture all item-level, scoring, or structural modifications made. The full EMIT and REMIT Fidelity Tools are available as Supplementary Material for further in-depth comparisons.*
